# A Smartwatch Step-Counting App for Older Adults: Development and Evaluation Study

**DOI:** 10.2196/33845

**Published:** 2022-08-10

**Authors:** George Boateng, Curtis L Petersen, David Kotz, Karen L Fortuna, Rebecca Masutani, John A Batsis

**Affiliations:** 1 Department of Management, Technology and Economics ETH Zurich Zurich Switzerland; 2 Geisel School of Medicine Dartmouth College Hanover, NH United States; 3 Division of Geriatrics and Palliative Care Icahn School of Medicine at Mount Sinai New York, NY United States; 4 Division of Geriatric Medicine School of Medicine University of North Carolina at Chapel Hill Chapel Hill, NC United States; 5 Department of Nutrition Gillings School of Global Public Health University of North Carolina at Chapel Hill Chapel Hill, NC United States; 6 The Dartmouth Institute for Health Policy & Clinical Practice Geisel School of Medicine at Dartmouth Hanover, NH United States

**Keywords:** step tracking, step counting, pedometer, wearable, smartwatch, older adults, physical activity, machine learning, walking, mHealth, mobile health, mobile app, mobile application, app, uHealth

## Abstract

**Background:**

Older adults who engage in physical activity can reduce their risk of mobility impairment and disability. Short amounts of walking can improve quality of life, physical function, and cardiovascular health. Various programs have been implemented to encourage older adults to engage in physical activity, but sustaining their motivation continues to be a challenge. Ubiquitous devices, such as mobile phones and smartwatches, coupled with machine-learning algorithms, can potentially encourage older adults to be more physically active. Current algorithms that are deployed in consumer devices (eg, Fitbit) are proprietary, often are not tailored to the movements of older adults, and have been shown to be inaccurate in clinical settings. Step-counting algorithms have been developed for smartwatches, but only using data from younger adults and, often, were only validated in controlled laboratory settings.

**Objective:**

We sought to develop and validate a smartwatch step-counting app for older adults and evaluate the algorithm in free-living settings over a long period of time.

**Methods:**

We developed and evaluated a step-counting app for older adults on an open-source wrist-worn device (Amulet). The app includes algorithms to infer the level of physical activity and to count steps. We validated the step-counting algorithm in the lab (counting steps from a video recording, n=20) and in free-living conditions—one 2-day field study (n=6) and two 12-week field studies (using the Fitbit as ground truth, n=16). During app system development, we evaluated 4 walking patterns: normal, fast, up and down a staircase, and intermittent speed. For the field studies, we evaluated 5 different cut-off values for the algorithm, using correlation and error rate as the evaluation metrics.

**Results:**

The step-counting algorithm performed well. In the lab study, for normal walking (*R*^2^=0.5), there was a stronger correlation between the Amulet steps and the video-validated steps; for all activities, the Amulet’s count was on average 3.2 (2.1%) steps lower (SD 25.9) than the video-validated count. For the 2-day field study, the best parameter settings led to an association between Amulet and Fitbit (*R*^2^=0.989) and 3.1% (SD 25.1) steps lower than Fitbit, respectively. For the 12-week field study, the best parameter setting led to an *R*^2^ value of 0.669.

**Conclusions:**

Our findings demonstrate the importance of an iterative process in algorithm development before field-based deployment. This work highlights various challenges and insights involved in developing and validating monitoring systems in real-world settings. Nonetheless, our step-counting app for older adults had good performance relative to the ground truth (a commercial Fitbit step counter). Our app could potentially be used to help improve physical activity among older adults.

## Introduction

Older adults are faced with an increased risk of developing multiple comorbid medical conditions, social isolation, and reduced physical function, which can lead to an increased risk of disability [1]. An inability to engage in activities of daily living may increase mortality risk and premature nursing home placement [2]. Hence, it is critical to encourage older adults with multimorbidity to engage in interventions that improve health, including physical activity. In fact, simple community-based walking programs and resistance-based programs [3] have effectively demonstrated reductions in the long-term risk of disability [4]. Even short bouts of walking can improve quality of life, physical function, and cardiovascular fitness in older adults [5].

Traditional consumer-based health-promoting endeavors, such as Silver Sneakers [6], have been scaled and widely disseminated across the United States. Randomized control trials have also shown the short- and long-term benefits of physical activity. However, sustained motivation continues to be a challenge for many individuals. Simple encouragement by clinicians may enhance engagement [7]. Yet, a study of accelerometry data demonstrated that only 7.6% of older adults aged 65 years meet Physical Activity Guidelines for Americans [8]. These pragmatic challenges demonstrate the need to overcome the barriers of traditional health promotion to enhance self-efficacy and behavioral change.

Older adults are the fastest-growing group of technology users; one survey suggested that 61% of older adults use smartphones [9], an increase from 23% in 2013 [10]. In fact, over 50% of older adults use social media [9]. Remote monitoring using fitness devices has now become ubiquitous in many countries where technology is readily available. In both consumer-based and academic-focused trials, it continues to be challenging to find a balance between clinical accuracy and ease of use. Current algorithms in consumer devices (such as Fitbit) are proprietary and often are not tailored to the group being evaluated in a clinical setting, such as older adults in free-living conditions, and have been shown to have wide error rates in such contexts [11,12]. A few smartwatch-based step-counting algorithms have been developed using data from young adults and validated in controlled settings only [13,14]. Matthies et al [15] developed a smartwatch step-counting app for older adults who use a walking frame equipped with wheels, which was evaluated outdoors, but only in a controlled setting, with 5 older adults. To the best of our knowledge, a smartwatch step-counting app for older adults has not been developed and validated in free-living settings over a long period of time with a large sample.

We previously developed GeriActive, an app that measures the daily activity levels (low, moderate, or vigorous) of older adults [16]. We aimed to develop and validate a smartwatch-based step-counting algorithm for older adults that runs as an app on the Amulet device. The Amulet is an open-source wrist-worn device that has been used for various mobile health studies, such as stress and physical activity monitoring [17,18]. The Amulet platform enables developers to write energy- and memory-efficient apps.

## Methods

### Study Overview

We validated the step-counting algorithm with older adults in the lab (using videorecordings as ground truth) and in 2 free-living studies (using the Fitbit as ground truth) lasting 2 and 12 days.

### Overview of Step Counter App

**Figure 1:**
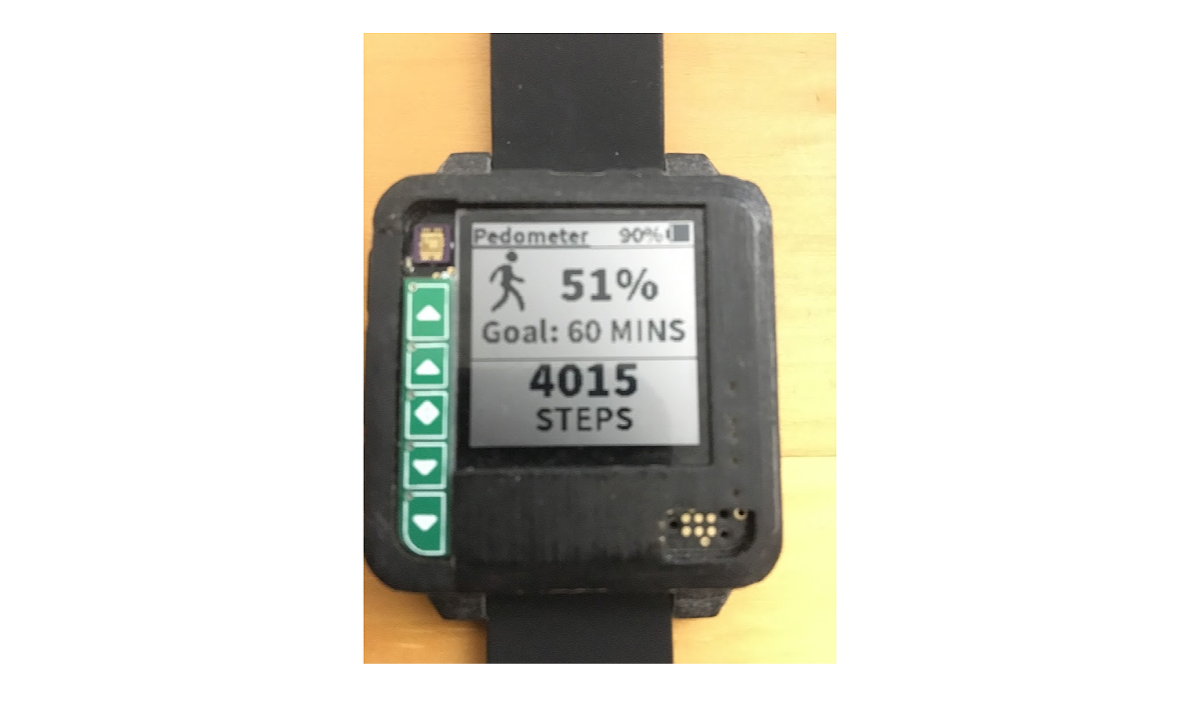
The Step Counter App with the step count displayed at the bottom.

Our step-counting Amulet app estimates the number of steps taken over the course of a day and displays the information on the Amulet screen, similar to the functionality of a pedometer or of other wearable devices (Figure 1). The app continuously estimates the number of steps for each 5-second window, updating the count viewed by the user. It uses a 2-step process: machine learning is used to determine if physical activity occurred in the most recent 5 seconds of data, and then, the number of steps is estimated by counting the number of peaks.

### Activity-Detection Algorithm

We used a linear support vector machine that classifies each 5-second window of accelerometer data into low, moderate, or vigorous activity [16]. We trained the algorithm on data collected from older adults who performed various physical activities: sitting, standing, lying down, walking, and running [16,19]. Our evaluation of the algorithm produced an accuracy of 91.7% using leave-one-subject-out cross-validation. If the output of the algorithm is moderate or vigorous, the step-detection algorithm is run to determine the number of steps in the 5-second window. This 2-phase approach was necessary to reduce false positives by preventing various actions such as random hand motions from being counted as steps.

### Step-Detection Algorithm

The step-detection algorithm estimates the number of steps in 5 seconds of acceleration data. The algorithm uses the magnitude of the acceleration of the 3-axis accelerometer of the Amulet. It is a 3-stage process consisting of zero-meaning, filtering, and peak detection, using an approach similar to that described in [13]. First, to ensure the data have a mean of zero, for each sample, we subtract the average of the preceding 20 data samples. Subsequently, a moving-average filter is applied, that is, each sample is replaced with the average of the 3 preceding samples. Finally, peaks in the filtered signal are identified by checking for change of slope. If the slope changes from positive to negative, and the peak value is above a certain threshold, then the peak is counted as a step. The cut-off threshold was initially empirically determined and then tuned.

### Ethics

Studies were approved by the Committee for the Protection of Human Subjects at Dartmouth College and the Dartmouth-Hitchcock Institutional Review Board (28905). All participants provided signed informed consent.

### Participants

Participants were recruited through the Center for Health and Aging at Dartmouth and primary care practices at Dartmouth-Hitchcock using flyers, listservs, and posters. This was a convenience sample; our results may not necessarily be applicable to other groups.

### Laboratory Study

Data for the development and evaluation of the step-detection algorithm were collected at our Dartmouth campus laboratory. Older adults (n=20, age ≥65 years) were asked to perform different types of walking (normal, fast, up and down a staircase, and intermittent) while wearing an Amulet. The Amulet collected raw acceleration data at a frequency of 20 Hz and logged the magnitude, which we later used to develop the step algorithm. The participants were videotaped. The video was independently reviewed to count steps by 2 individuals independently (JAB, RKM) and any differences were later reconciled. We used these data for the preliminary development of the step-detection algorithm and evaluated the algorithm using the error rate (the percentage difference between the algorithm’s estimated step count and the ground-truth step count measured from the video).

### 2-Day Field Study

We conducted a 2-day field study in which older adults (n=7, age ≥65 years) wore an Amulet device (running our step counter app) and a Fitbit Flex 2 device (Fitbit Inc) on the same wrist for 2 days. We compared each participant’s step count estimated by their Amulet (exploring 5 different cut-off values) with their step count reported by the Fitbit (downloaded from the individual’s research-based Fitbit account). The error rate between Fitbit’s steps and Amulet’s steps for each of the 5 peak cut-off values was computed.

### 12-Week Field Study

We conducted a field study (2 cohorts, 12 weeks each) to test the step-detection algorithm with the target population—older adults with obesity. This study was a subset of a larger study that evaluated the impact of a combined weight loss and exercise intervention in older adults with obesity [20]. The goal of this analysis was to compare the Amulet’s step-count estimate with the Fitbit’s step count over a long period in real-world settings. Participants (Table 1) from both rounds wore an Amulet and Fitbit on the same wrist for 12 weeks. The Amulet logged the summary steps on an SD memory card hourly and at the end of each day. A research assistant copied the data from each participant’s SD card on a biweekly basis. The Fitbit logged a summary of each day’s step count (computed by a proprietary algorithm) to the Fitbit app on the participant’s Android tablet, which uploaded the data to the Fitbit servers; we subsequently used the Fitbit research API to download participants’ data. After 10 weeks of monitoring data from the first cohort, we modified the step-detection peak cut-off value to minimize the error rate relative to ground-truth step count data from the Fitbit.

**Table 1:** Participant characteristics.

Characteristic			Value (n=16)
**Age (years)**			
	Mean (SD)	74.1 (5.6)	
	Range	66-87	
**Sex, n (%)**			
	Male	4 (25)	
	Female	12 (75)	
**Marital status, n (%)**			
	Married	7 (44)	
	Divorced	8 (50)	
	Widowed	1 (6)	
**Smoking history, n (%)**			
	None	13 (81)	
	Formerly smoked	3 (19)	
**Education, n (%)**			
	High school	2 (12)	
	Some college	5 (31)	
	College degree	3 (19)	
	Postcollege degree	6 (38)	
Weight (kg), mean (SD)			97.06 (18.2)
Body mass index (kg/m^2^), mean (SD)			36.8 (4.9)
Multimorbidity, n (%)			14 (87)
**Comorbidities, n (%)**			
	Diabetes	2 (12)	
	Fibromyalgia	1 (6)	
	High cholesterol	6 (38)	
	Hypertension	9 (56)	
	Osteoarthritis	6 (38)	

### Statistical Analysis

We calculated the mean with standard deviation for continuous measures and count with percentage for categorical measures. Although participants were instructed to wear the Amulet and Fitbit simultaneously, not all participants did so the entire time. We thus limited the data to days for which it was safe to assume that they were worn for nearly the same amount of time. The Amulet was able to detect if it was worn each hour, so we considered the Amulet to be worn if the Amulet was worn for 75% of a 15-hour day (675 minutes); the Fitbit only reported daily step count, so we considered the Fitbit to be worn if the step count was greater than 100. We selected these parameters from an understanding of the distribution of wear time over the course of a 24-hour day and the distribution of steps expected for this population per day [21]. Comparisons between Amulet and Fitbit were limited to data from days for which both were worn. We conducted bivariate linear regression to compare the association between Amulet and video-counted steps during various activities (laboratory study) and between Amulet and Fitbit steps (2-day and 12-week field studies). We compared Amulet steps to Fitbit steps using percentage difference (difference between Amulet and Fitbit steps divided by Fitbit steps).We used Bland-Altman plots to compare the agreement between Amulet and Fitbit steps. For our analysis, we defined significance as *P*<.05.

## Results

### Laboratory Study

**Figure 2:**
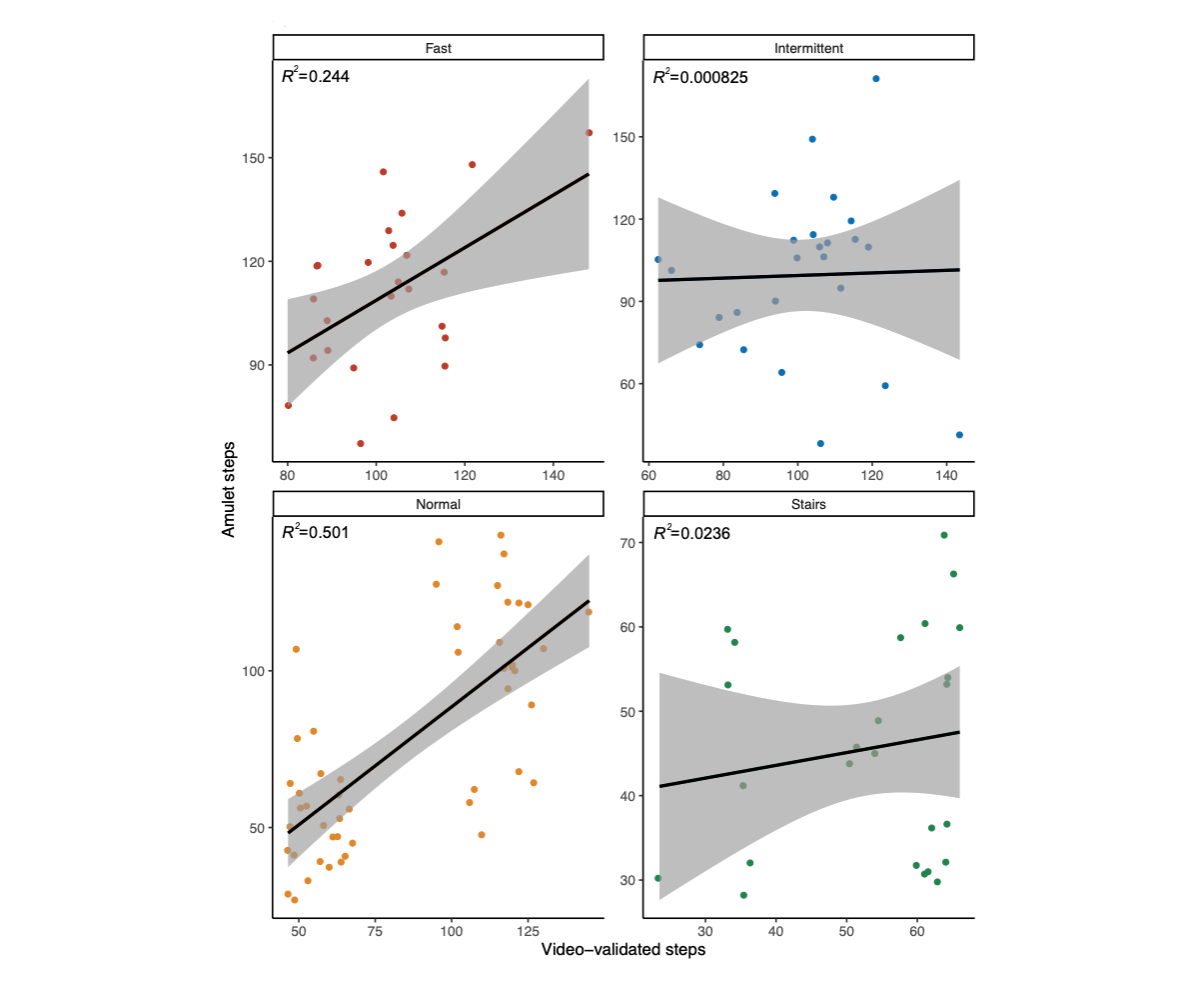
Association between Amulet-estimated steps and video-validated steps.

There was a strong linear association when participants walked normally (Figure 2; Table 2). For normal walking, the Amulet step-detection algorithm undermeasured the number of steps taken by an average of 6.7 steps (SD 32.6). Across all activities, the Amulet was on average 3.2 steps lower (SD 25.9) or 2.1% (SD 31.9%) lower than video-validated steps (Figure 2). The distribution was slightly positively skewed.

**Table 2:** Step count for different walking activities.

Activity	Video-validated, n (%)	Amulet, n (%)	Percentage error
Fast	102.62 (14.7)	110.72 (22.7)	8.53 (20.02)
Intermittent	101.02 (18.7)	99.48 (30.8)	1.71 (33.28)
Normal	84.9 (31.9)	77.14 (33.9)	–6.68 (32.55)
Stairs	52.76 (13.4)	45.52 (13.2)	–7.55 (36.97)

### 2-Day Field Study

**Figure 3:**
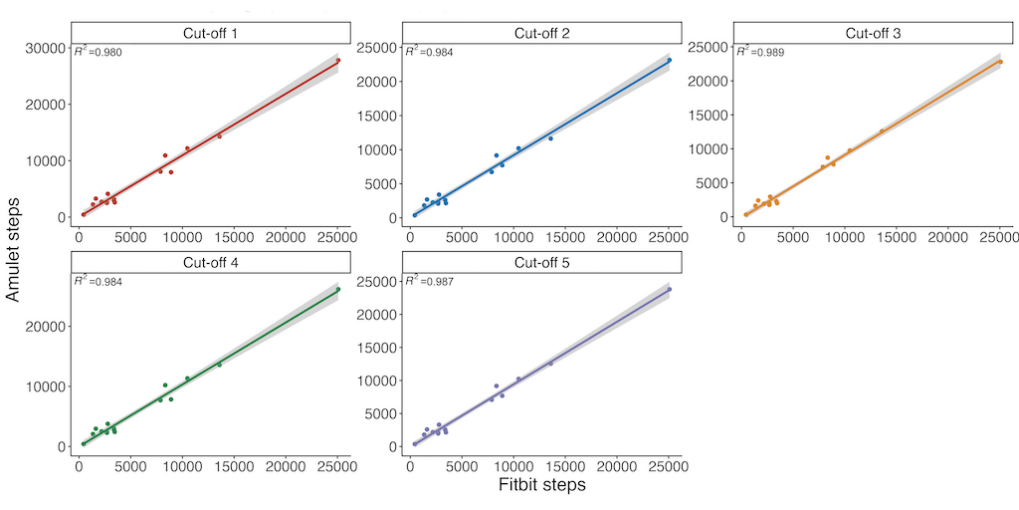
Association between Amulet and Fitbit steps for different cut-off values: 2-day field study.

**Figure 4:**
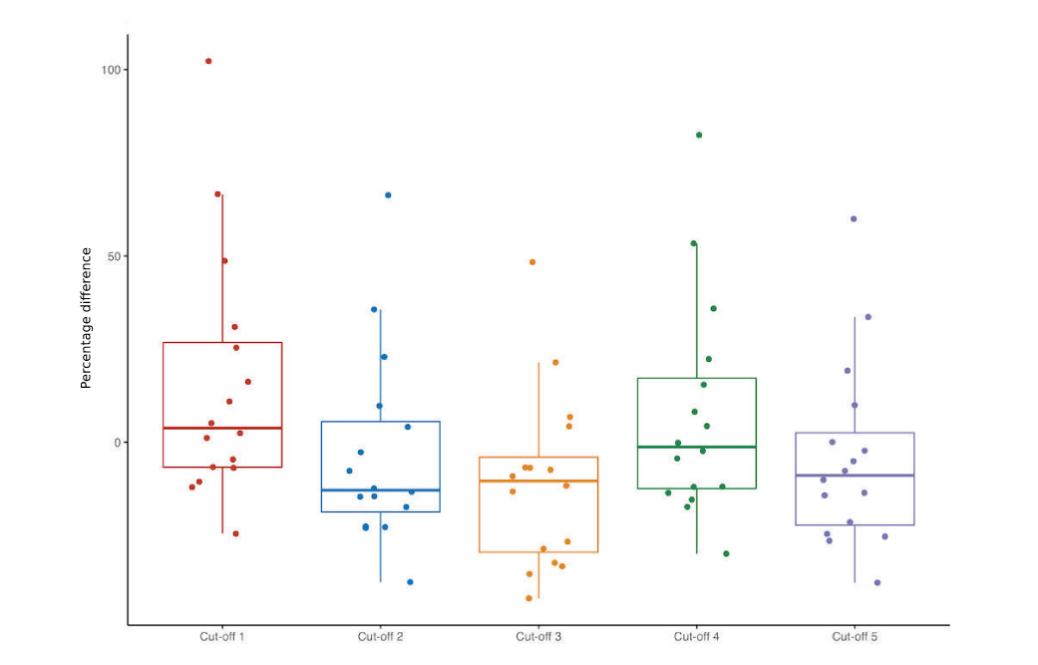
Distribution of percentage difference between Amulet and Fitbit steps by cut-off threshold.

We discarded 1 participant’s data because the data indicated the devices had not been worn much (step counts were less than 350 per day). The associations between each participant’s daily step counts reported by the Amulet and Fitbit were high for all cut-off thresholds (Figure 3). The third cut-off threshold had the highest association between Amulet and Fitbit steps (*R*^2^=0.989). Cut-off threshold number 2 had the smallest mean percentage difference between Amulet and Fitbit (–3.1%, SD 25.1) of all cut-off thresholds (threshold 1: mean 15.27%, SD 33.19; threshold 3: mean –10.77, SD 23.43; threshold 4: mean 7.18, SD 29.28; threshold 5: mean –4.11, SD 24.94) (Figure 4).

### 12-Week Field Study Results

**Figure 5:**
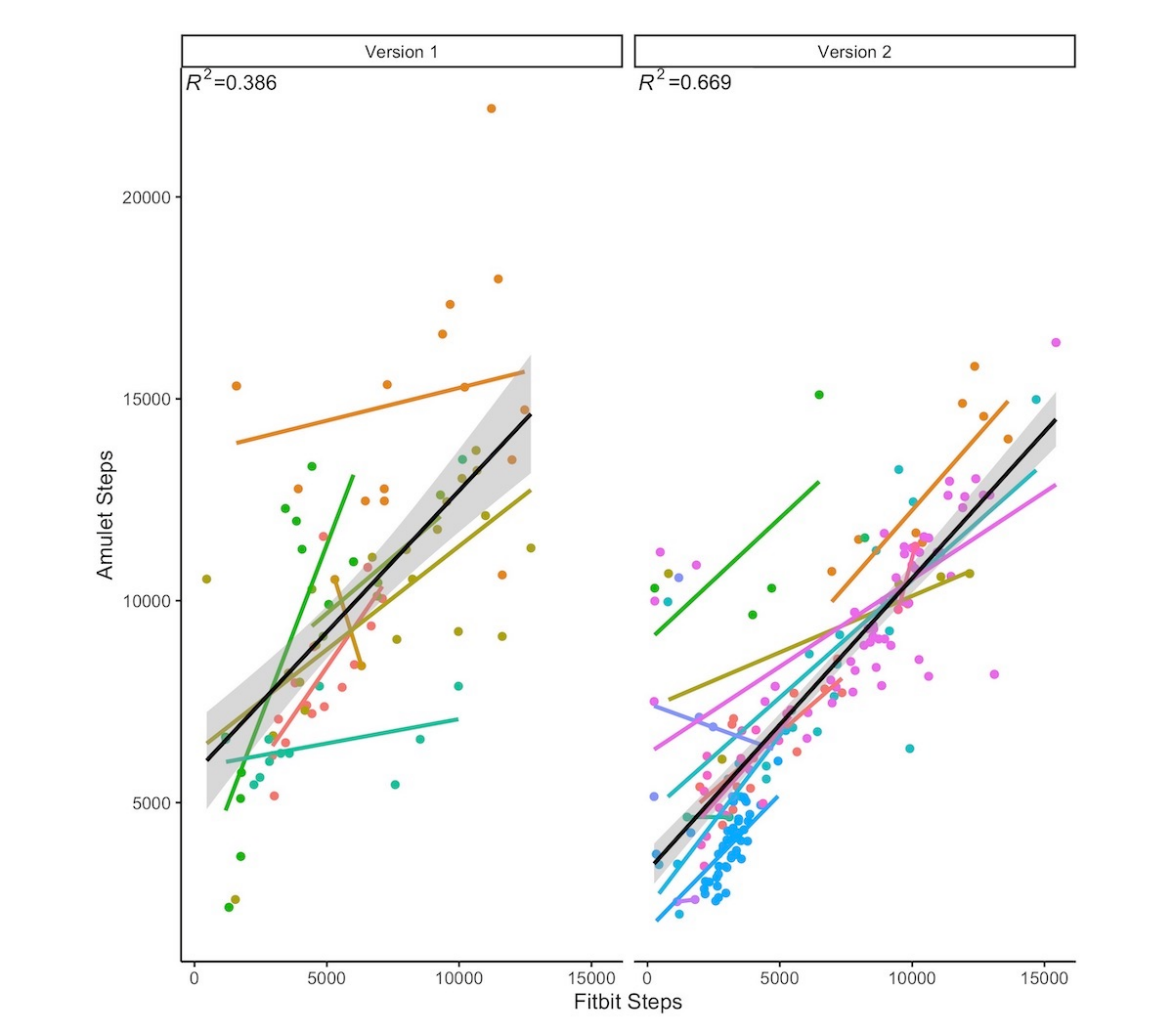
Association between Amulet and Fitbit steps by algorithm version. Each line is a linear regression for each participant, colored separately, with the overall linear regression in black. Version 1 represents the app used during the first 10 weeks of cohort 1, and version 2 represents the app used during the final 2 weeks of cohort 1 and all 12 weeks of cohort 2.

**Figure 6:**
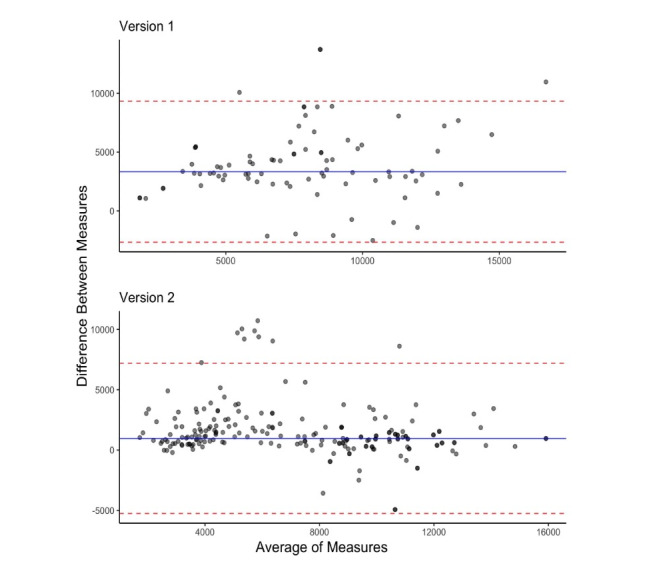
Bland-Altman plots of Amulet and Fitbit step measures by algorithm version. The blue line represents the mean difference between Amulet and Fitbit steps per day. Each red line represents 2 SD of the difference. Version 1 represents the app used during the first 10 weeks of cohort 1, and version 2 represents the app used during the final 2 weeks of cohort 1 and all 12 weeks of cohort 2.

Across both 12-day field studies, there were 297 participant-days for which both the Fitbit and Amulet had been worn. Cohort 2 used the modified app. For the first cut-off threshold (version 1), we recorded 86 participant-days; the average Fitbit step count was 5797 steps (SD 3296), and the average Amulet step count was 9780 steps (SD 3719). For the second cut-off threshold (version 2), we recorded 211 participant-days; the average Fitbit step count was 6415 per day (SD 3751), and the average Amulet step count was 7956 per day (SD 3324). The association between Fitbit steps and Amulet steps improved (first cut-off threshold: *R*^2^=0.386; second cut-off threshold: *R*^2^=0.669) (Figure 5). There was improved agreement between both measures with the second cut-off threshold (Figure 6). There were 9 observations by 5 unique participants with differences 2 standard deviations higher than the combined difference mean. These observations had an average Fitbit and Amulet step count of 1373 (SD 1988) and 10,689 (SD 1971), which suggested that participants may have taken their Fitbits off at some point during the day before removing their Amulet.

## Discussion

We found that an open-source platform and algorithm developed for older adults can capture daily step counts with reasonable accuracy. Our findings demonstrate the importance of an iterative process in algorithm development before field deployment. First, our lab-based data provided confidence in the algorithm’s step estimates, making a case for a real-world evaluation. We then tested the algorithm in a 2-day field study before full-scale deployment. The step estimates from our algorithm were highly correlated with the step counts from the Fitbit for all peak cut-off thresholds, with low error rates. These results provide evidence that the algorithm worked well in free-living conditions, albeit for a short duration. We used the cut-off threshold with the lowest error rate for the subsequent field studies conducted over longer periods.

As with any user study, there are differences between field-based conditions and those in laboratory settings or short-duration studies. The poorer results in the 12-week study (cohort 1) may have been a result of the differences between the populations used for developing and evaluating the algorithm (older adults vs older obese adults). Older adults with obesity have a higher degree of comorbid conditions [22], along with differences in stride length, cadence, and gait [23], which may impact either algorithm. Additionally, the longer time period (12 weeks vs 2 days) could have allowed the occurrence of a greater number of confounding situations, such as both devices not being worn at all times or one device being off while the other was on. These results make a strong case for developing and refining algorithms with data from the target population and evaluating algorithms in the conditions for which they were designed.

Based on our observations in the first 10 weeks of cohort 1, we modified the peak cut-off threshold to minimize the error rate and evaluated version 2 of the algorithm in a study of the same duration with different participants from the same target population (cohort 2). Version 2 exhibited better performance in terms of correlations and error rates.

One limitation of this work is that we used the Fitbit as ground truth. Given that the Fitbit device (and its proprietary algorithm) was not specifically developed for this population (older adults with obesity), it is difficult to say whether our algorithm performed better or worse than Fitbit’s algorithm relative to the ground truth. The ideal ground truth would have been to use video recording, as we did in the lab study, but videorecording is not feasible for field studies due to privacy limitations. Hence, a device such as the Fitbit was the best compromise.

Although Fitbit outputs have been shown to have high correlations with those of other step-counting devices when used with older adults in free-living settings, results have varied depending on the specific Fitbit device used, device placement (wrist vs waist), and the comparison device used [24-27]. We hypothesized that the Fitbit would underestimate the steps of older adults in comparison to the Amulet, because the Fitbit was developed using data from younger adults, and older adults move more slowly [28]. Thus, we expect the overall true step count to be higher than Fitbit’s estimate. Hence, we settled for the case where our algorithm overestimated the steps but was highly correlated with Fitbit’s estimate. In addition, the Fitbit data were captured daily, whereas Amulet data were captured hourly. Had hourly data been available from both, we could have performed a fine-grained comparison between the algorithms. Finally, it was not possible to get a good sense of wear time from the Fitbit as we did in the Amulet. We could only use the Amulet’s wear time estimate and a minimum Fitbit daily step count of 100 steps as the threshold for being worn.

Because we were evaluating predominantly intraperson variability (ie, the purpose was not to evaluate the impact of any intervention), we did not measure baseline characteristics of the participants (eg, disease regarding walking behavior, such as Parkinson disease, musculoskeletal disorders). Future studies should determine whether such characteristics could have an impact on our results.

The use of an open-source system, such as the Amulet, highlights researchers’ ability to develop algorithms that are tailored and trained for a target population such as older adults. With the constant iteration of consumer devices and algorithms, it is difficult to ensure precision and accuracy for groups that need to be more physically active, such as older adults. Hence, it is important to develop and examine products that can meet their needs. Providing imprecise or inaccurate information on physical activity could undermine the motivation of this population to increase their physical activity. We recommend that future work demonstrate validity of algorithms in these populations and identify situations where data collection can be the most clinically relevant and actionable.

Our step-count algorithm performed well in comparison with Fitbit, with high correlations and low error rates. Overall, this work highlights various challenges and insights involved in developing and validating monitoring systems in real-world settings.

## Abbreviations

API: application programming interface
